# Endoscopic Endonasal Approach in the Management of Rathke’s Cleft Cysts

**DOI:** 10.1371/journal.pone.0139609

**Published:** 2015-10-16

**Authors:** Domenico Solari, Luigi Maria Cavallo, Teresa Somma, Carmela Chiaramonte, Felice Esposito, Marialaura Del Basso De Caro, Paolo Cappabianca

**Affiliations:** 1 Department of Neurosciences and Reproductive and Odontostomatological Sciences, Division of Neurosurgery, Università degli Studi di Napoli "Federico II", Naples, Italy; 2 Department of Neurosciences, Division of Neurosurgery, Università degli Studi di Messina, Messina, Italy; 3 Department of Biomorphological and Functional Sciences, Division of Pathology and Cytopathology, Università degli Studi di Napoli Federico II, Naples, Italy; Emory University School of Medicine, UNITED STATES

## Abstract

**Objective:**

Rathke’s cleft cysts (RCCs) are quite uncommon sellar lesions that can extend or even arise in the suprasellar area. The purpose of this study is to evaluate the effectiveness of both standard and extended endoscopic endonasal approaches in the management of different located RCCs.

**Methods:**

We retrospectively analyzed a series of 29 patients (9 males, 20 females) complaining of a RCC, who underwent a standard or an extended endoscopic transsphenoidal approach at the Division of Neurosurgery, Department of Neurosciences and Reproductive and Odontostomatological Sciences, of the Università degli Studi di Napoli "Federico II”. Data regarding patients’ demographics, clinical evaluation, cyst characteristics, surgical treatments, complications and outcomes were extracted from our electronic database (Filemaker Pro 11, File Maker Inc., Santa Clara, California, USA).

**Results:**

A standard transsphenoidal approach was used in 19 cases, while the extended variation of the approach in 10 cases (5 purely suprasellar and 5 intra-suprasellar RCC). Cysts contents was fully drained in all the 29 cases, whilst a gross total removal, that accounts on the complete cyst wall removal, was achieved in an overall 55,1% of patients (16/29), specifically 36,8% (7/19) that received standard approach and 90% (9/10) of those that underwent to extended approach. We reported a 56.2% of recovery from headache, 38.5% of complete recovery and 53.8% of improvement from visual field defect and an overall 46.7% of improvement of the endocrine functions. Postoperative permanent DI rate was 10.3%, overall post-operative CSF leak rate 6.9%; recurrence/regrowth occurred in 4 patients (4/29, 13.8%), but only one required a second surgery.

**Conclusion:**

The endoscopic transsphenoidal approach for the removal of a symptomatic RCC offers several advantages in terms of visualization of the surgical field during both the exposure and removal of the lesion. The “extended” variation of the endoscopic approach provides a direct access to the supradiaphragmatic space, allowing adequate view and room for the safe removal of selected supradiaphragmatic RCCs, regardless of the sellar size (even a not enlarged sella), and provides a higher likelihood of preserving normal pituitary tissue and functions.

## Introduction

Rathke’s cleft cysts (RCCs) are benign cystic lesions forming out of remnant cells of the craniopharyngeal duct within Rathke’s pouch. In routine autopsies, they are encountered in 13–33% of normal pituitary glands and, actually, account for the 2–9% of all the intracranial tumors removed via a transsphenoidal approach [1–15].

Histologically, RCCs consist of a single layer or pseudostratified epithelium with an underlying layer of connective tissue. The epithelium can be made of ciliated, goblet, squamous or basal cells. The cyst contents can be made of mucinous, gelatinous or caseous cystic fluid, which presents different imaging characteristics. The embryological development of Rathke cleft cysts has been already described elsewhere [16–20]. The most accepted hypothesis states that a rostral outpouching of the primitive oral cavity in the 3^rd^ or 4^th^ week of gestation meets a downward projection from the diencephalon: these two structures give rise to the anterior lobe, pars tuberalis and pars intermedia of the pituitary gland [16, 17, 19]. During the formation of the adenohypophysis and neurohypophysis a cleft remains patent in the pars intermedia region, i.e. the Rathke cleft; when this cleft fails to regress, a Rathke cleft cyst develops. Other pathogenic theories account on an endodermal origin [21] or reverse metaplasia of pituitary cells [19].

These lesions can remain located within the sella, extend into the suprasellar space or arise as purely suprasellar lesions. In the majority of cases RCCs remain asymptomatic, however a significant growth can determine mass effect on the surrounding structures causing endocrinological and/or neurological dysfunction—seldom resembling clinical features of pituitary apoplexy—and, eventually, requires surgical removal [2, 5–8, 22–27]. The best surgical strategy ought to be adopted, varies according both to clinical status and cyst location. Along the years, with the flourishing of the endoscopic endonasal surgery for pituitary lesion, this technique has been advocated for the treatment of different sellar and suprasellar lesions, including the Rathke’s cleft cyst [12–14, 28–30]. More recently, the definition of the so-called endoscopic endonasal “extended” approach granted access to the suprasellar area, thanks to the additional removal of the tuberculum sellae and posterior portion of the sphenoidal planum: indeed, the use of this corridor has rendered amenable the management of purely suprasellar lesions, traditionally removed via transcranial route only.

In this study we report the retrospective analysis of our series of RCCs in which both standard and “extended” endoscopic endonasal approaches have been used to manage such lesions. The aim of the present report is to evaluate the effectiveness of the endoscopic endonasal procedures also in regards to different approaches and lesion location.

## Materials and Methods

### Population and study design

This study was approved by the institutional review board of the School of Medicine of Università degli Studi di Napoli Federico II, which waived the necessity for informed consent due to the retrospective nature of the study. Written informed consent was obtained from the patients prior than any invasive clinico-diagnostic and surgical procedure; indeed, it was obtained for the eventual publication—for scientific purpose—of any patient records/information anonymously.

Between January 1997 and January 2014, 29 consecutive patients out of 1265 endoscopic procedures- representing the 2,3% (29/1265) of the series were complaining of a RCC and underwent an endoscopic endonasal surgery at our Department (Division of Neurosurgery of the Università degli Studi di Napoli Federico II, Naples—Italy).

Rathke’s cleft cyst was suspected when a midline, homogeneous fluid lesion—spontaneously hypointense in T1 and hyperintense in T2 weighted images—not enhancing after GAD injection, was observed at MRI.

Diagnosis was confirmed in each case as per hystopathological report upon the evidence of typical pseudo-stratified columnar ciliated cells defining the epithelium of the cyst wall, associated with the presence of mucinous fluid content [18].

Out of 29 patients, 20 were female (68,96%)—4 (20%) in post-menopausal age and 16 (80%) in premenopausal age—and 9 male; the mean overall age was 39,5 years (range 15–60 years).

Visual function was evaluated by automatic computerized perimetry, as well as visual acuity testing, before and after surgery. The subjective features, site and severity of headaches were recorded, in accordance to patient description. Endocrinological assessment was defined by measurements of the basal and functional pituitary hormone assays (PRL; GH; IGF–1, FSH; LH, testosterone, estradiol, FT4, cortisol, TSH and ACTH). The condition of panhypopituitarism was defined as the defect of three or more hormonal axes, whereas partial hypopituitarism was ruled out in case of two axes deficiencies. According to tumor location patients were divided, as described by Potts [9], into three groups: type I: intrasellar, type II: intra and supra or infrasellar and type III: suprasellar.

All the data were retrieved from our electronic database (Filemaker Pro 11, File Maker Inc., Santa Clara, California, USA).

### Surgical technique

Surgical treatment of the RCCs was performed via a “standard” endoscopic endonasal approach in 19 cases, whereas 10 patients underwent a transtuberculum transplanum “extended” approach. This latter has been used in all the 5 cases of suprasellar RCCs and in 5 cases of intra-suprasellar lesions (see Table 1).

**Table 1:** Surgical approach according to cyst location.

Location	Standard approach (19)	Extended approach (10)
Intra-sellar	3 (15.7%)	none
Intra and supra-sellar	16 (84.2%)	5 (50%)
Supra-sellar	none	5 (50%)

Concerning the tumor location, 3 patients had purely intrasellar lesion (type I), 21 patients had an intrasellar cyst with suprasellar extension (type II), whereas 5 patients had a completely suprasellar cyst (type III).

The procedure was performed using a rigid 0-degree endoscope, 18 cm in length and 4 mm in diameter (Karl Storz Endoscopy, Tuttlingen, Germany), as the sole visualizing tool. The use of 30°-45° angled endoscopes was reserved to explore large intra-suprasellar post-surgical tumor cavities. The surgical procedures were run according to the techniques already described in previous publications [29, 31–33]. However, there were significant differences in the surgical management of the RCC with an intra or intra-suprasellar location and those with a purely suprasellar location, so that we will analyze relative peculiar features separately. Besides, as it revealed as major issue of this kind of surgery, especially during “extended” approaches, we briefly describe the different techniques of reconstruction used and accordingly defined 4 groups in the analysis of the series: 1) intradural filling with intradural closure; 2) intradural filling with extradural closure; 3) intradural repair with intra-extradural gasket seal/grandma’s cap closure" [34]; 4) intradural repair with intra-extradural gasket seal/grandma’s capclosure and pedicled nasoseptal flap [35, 36].

All patients received an intradural filling, consisting of dead space filling with fibrin glue and/or autologous fat and/or dural substitute in layers, in order to protect the suprasellar cistern or seal the arachnoid. The extradural closure consisted of the positioning of one or multiple dural substitute layers in the extradural space. Closure with a buttress, also known as the gasket seal or grandma’s cap technique [34] was performed with a dural substitute wedged in the intra or extradural space by a solid material (synthetic material or autologous bone) [37, 38].

### Intra-suprasellar RCC

**Fig 1:**
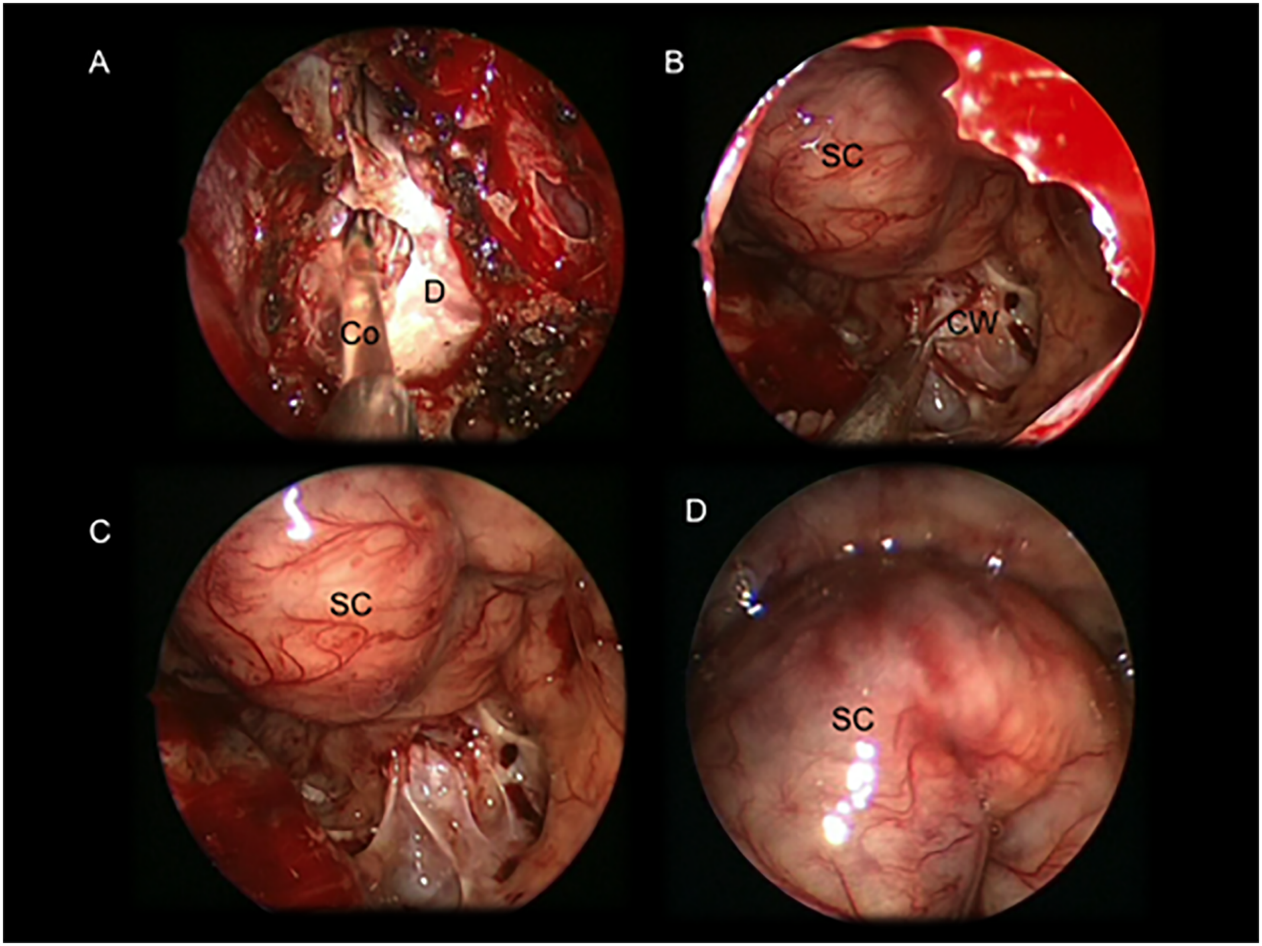
Intraoperative images showing an intra and suprasellar Ratkhe’s Cleft Cyst removed via a standard endoscopic endonasalapproach. (A) colloid suctioning after dural opening; (B: cyst wall removal (C) and (D) intrasellar view after the cyst wall removal. Suprasellar cistern covered by the stratified pituitary gland. *Co*: colloid; *CW*: cystwall; *D*: dura mater; *SC*: suprasellar cistern.

**Fig 2:**
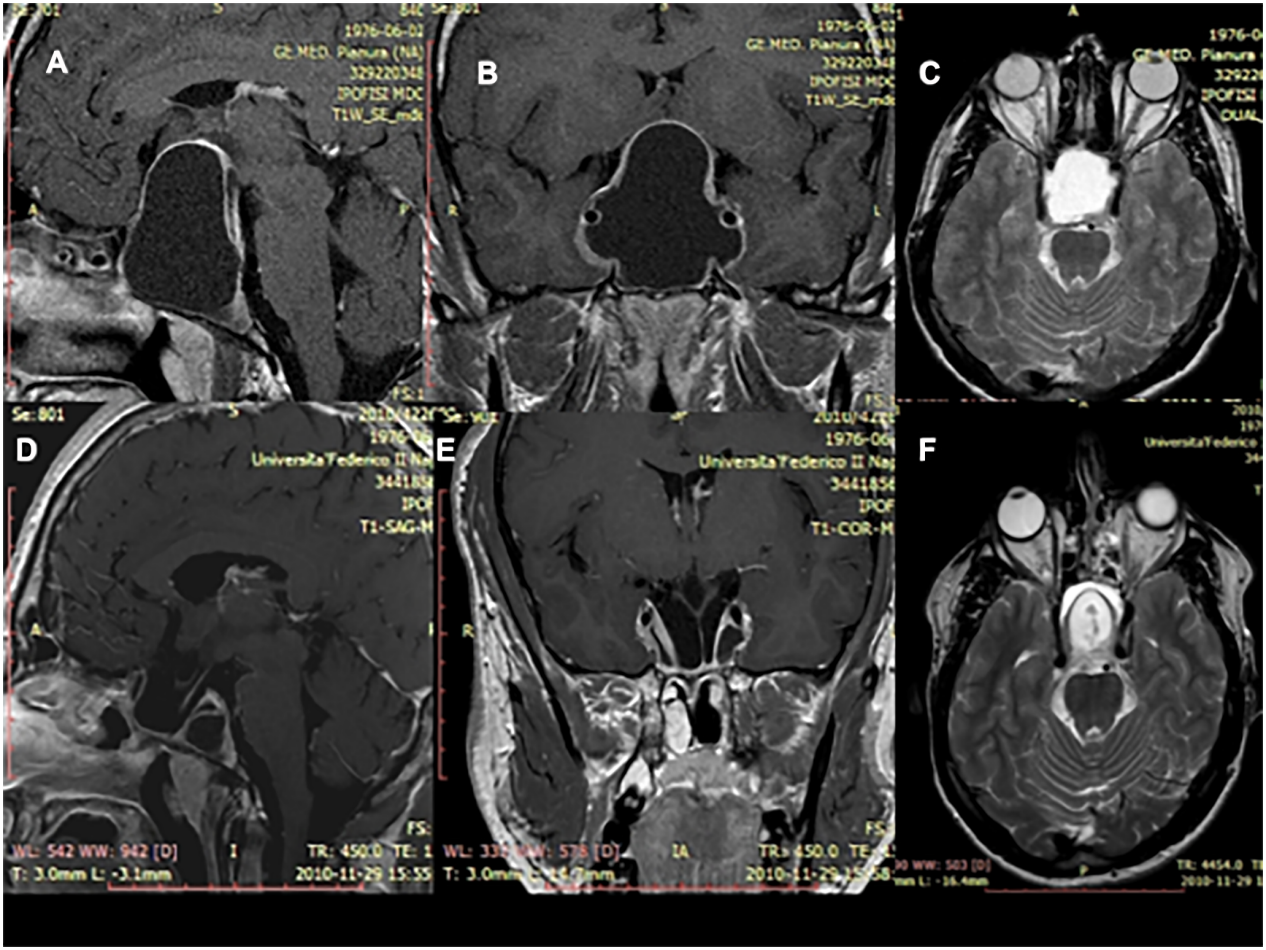
MRI scan after gadolinium showing an intra and suprasellar Rathke’s Cleft Cyst before and after the surgical removal via a standard endoscopic endonasal approach (case showed in the Fig 1). (A-B) Sagittal and a coronal T1-weighted scans of the lesion before being removed. The colloid has a hypointense signal and the cyst wall has post contrast enhancement. These features do not define typical aspect of RCC, whose differential diagnosis with sellar arachnoid cysts could be often challenging. (C) Axial T2-weighted scan of the lesion showing the colloid with a hyperintense signal. (D-E) Sagittal and a coronal T1-weighted scans and (F) axial T2-weightedscan at the three months postoperative MRI showing the cyst removal. It is possible to identify the decompression of the optic chiasm and the pituitary stalk.

The nasal and sphenoidal steps of the procedure are performed following the same principles of the standard pituitary approach for pituitary adenomas: binarial 3–4 hands technique is usually adopted; as for standard pituitary surgery, no middle turbinate is routinely removed in both nostrils, they are simply lateralized with an elevator and are pull back at the end of the procedure. In purely intrasellar RCC sellar floor is extensively removed down to the clival recess to grant a proper maneuverability of the surgical instruments inside the sella. In case of an enlarged sella, it can be useful to preserve a good extradural plane undermining bony edges, in order to allow an effective extradural closure of the sellar floor in case of intra-op CSF leak. Dura is opened as per accessing the cyst, thus avoiding normal pituitary gland injury: this could be particularly relevant in those cases of intra or intra-suprasellar RCC, where usually the adenohypophysis is pushed anteriorly by the cyst (originated from the pars intermedia of the pituitary gland). Though, the cyst is entered and emptied and any floating part of the cyst wall is taken out and, as the residual cavity is wide enough, the endoscope is inserted (Figs 1 and 2). Eventually, the so-called “diving technique” is realized by continuous irrigation through the irrigation sheath [39]. This permits the removal of any colloid remnant tightly adherent to the cyst wall and, eventually, the detachment of any wall fragments off the adenohypophysis and/or the diafragma sellae. In case of cyst wall tightly adherent to the pituitary tissue, dissection maneuvers are limited to avoid any postoperative impairment of the pituitary function. At the end of the procedure no sellar closure is performed, unless an intra-operative CSF leak occurred [38].

### Suprasellar RCC

**Fig 3:**
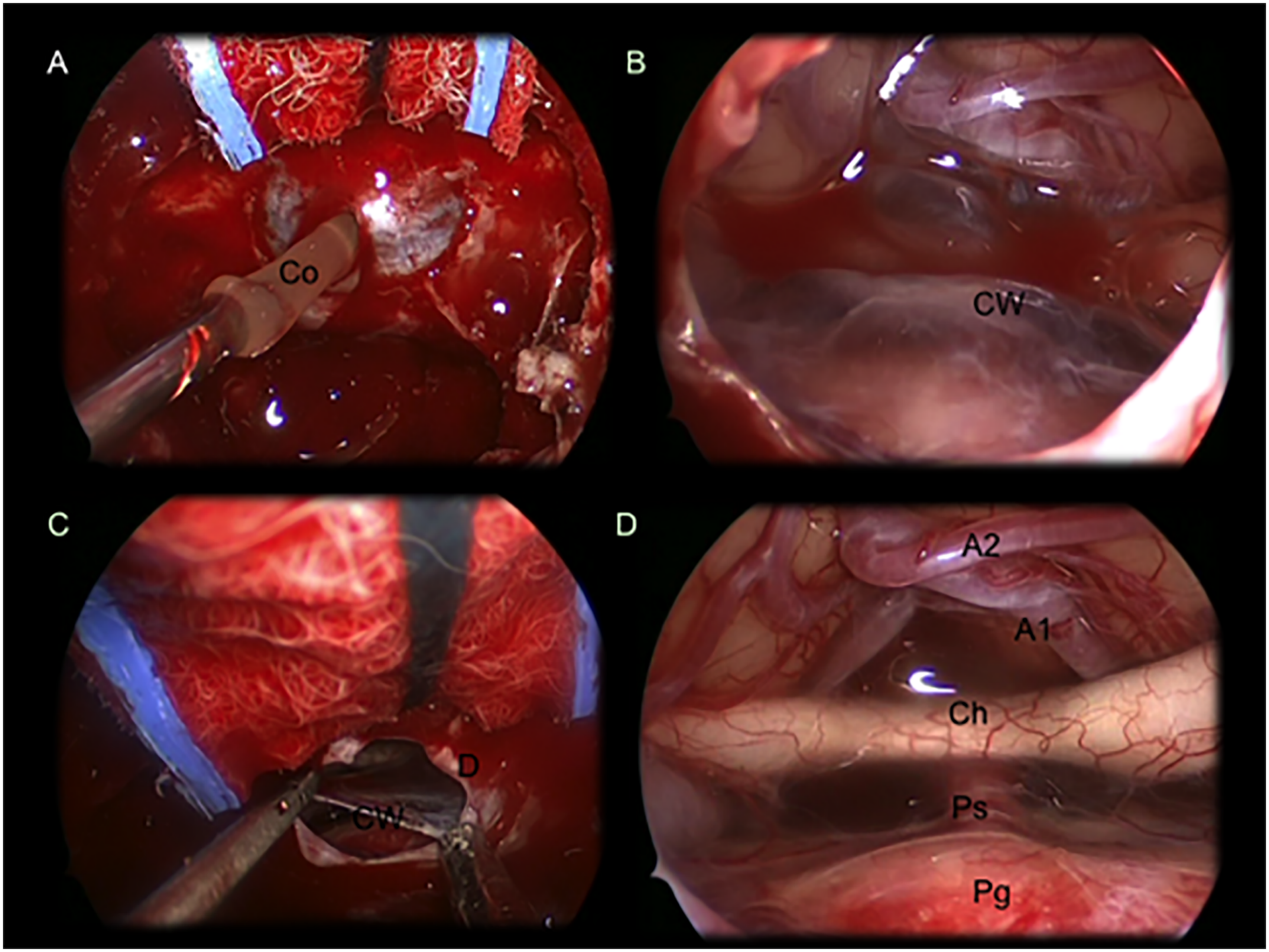
Intraoperative images showing a suprasellar Ratkhe’s Cleft Cyst removed via an extended endoscopic endonasalapproach. (A) colloid suctioning after dural opening and exposure of the cyst’s wall (B)Imagine showing the cyst’s wall covering the neurovascular structures of the suprasellar area. (C) cyst wall removal with a forceps and aspirator. (D) after cyst wall removal it is possible to identify: A1 and A2; optic chiasm with optic nerves; pituitary stalk and gland. *Co*: colloid; *CW*: cystwall; *D*: dura mater; *Ch*: optic chiasm; *Ps*: pituitary stalk; *Pg*: pituitary gland; *A1*: A1 segment of the anterior cerebral artery; *A2*: A2 segment of the anterior cerebral artery.

**Fig 4:**
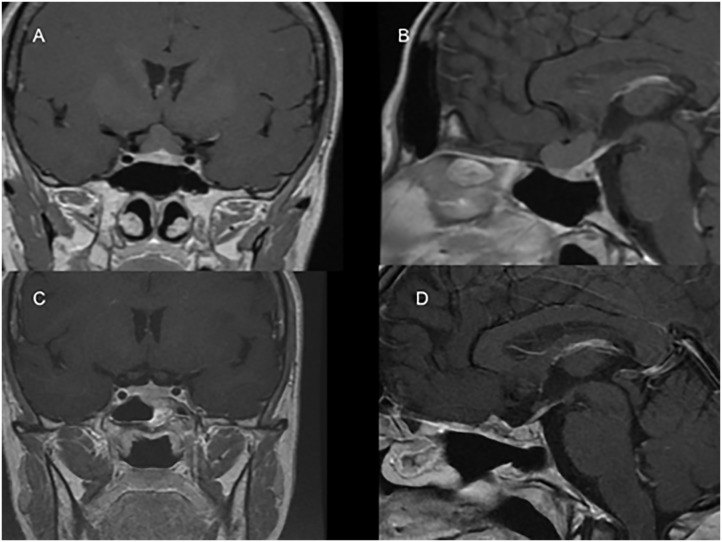
MRI scan showing a purely suprasellar Rathke’s Cleft Cyst before and after the surgical removal via an endoscopic endonasal approach (case showed in Fig 3). (A-B) Sagittal and coronal T1 weighted scans showing the lesion before being removed. The colloid has an isointense signal. (C-D) Sagittal and coronal post-gad scans after three months showing the cyst removal. It is possible to identify the optic chiasm the pituitary stalk and the normal pituitary gland.

In purely suprasellar RCC the sellar cavity is usually not enlarged and an endoscopic endonasal transtuberculum/transplanum approach is required to access the suprasellar area. As described elsewhere [28–32, 40], the approach is realized through both nostrils with a middle turbinectomy on one side, resection of the posterior portion of the nasal septum and a wider anterior sphenoidotomy. Owing that RCCs content is manly fluid, large bone opening over the planum sphenoidale is usually not required and extensive drilling at the level of the medial opto-carotid recess or over the planum sphenoidale is not mandatory [41]. The RCC is clearly identified after the dural opening with the pituitary stalk, often dislocated on one side. While the anterior part of the cyst wall is surrounded by the arachnoid of the suprasellar cistern, the remaining part of the cyst wall can be attached to the pituitary stalk, the superior hypophyseal arteries and/or the optic chiasm(Figs 3 and 4). In these cases it is of utmost importance to avoid tractions in order to prevent injuries to these structures. Bimanual dissection is performed according to the rules of microsurgery: one surgeon works bimanually to dissect and remove the cyst wall, while a second surgeon drives dynamically the endoscope.

## Results

### Signs and symptoms

Upon hospital admission, the most common presenting signs in our series were respectively headache, visual field defects (VFDs) and hormonal defects.

Chronic headache was reported in 55,17% (16/29) of cases, 44,83% (13/29) of patients presented visual function impairment, i.e. visual field defect. Of them 5 patients (38.4%) presented bilateral quadrantopia, 4 patients (30.8%) bilateral hemianopia and 4 other patients (30.8%) a monolateral quadrantopia. 51,72% (15/29) of the cases complained of pituitary dysfunctions: namely 7 cases of hyperprolactinemia, 5 patients with partial hypopituitarysm and 3 with panhypopituitarysm; we reported 1 single case of diabetes insipidus. All the data concerning preoperative endocrinological and visual status are summarized in Table 2.

**Table 2:** Preoperative clinical status according to cyst location.

Preoperative Endocrinological Status	Intrasellar(3)	Intra and suprasellar(21)	Suprasellar(5)	Total(29)
Normal	0 (0%)	11 (52.3%)	3 (60%)	14 (48.2%)
Hyperprolactinemia	1 (33.3%)	4 (19%)	2 (40%)	7 (58.6%)
Partial hypopituitarism	2 (66.6%)	3 (14.2%)	none	5 (17.2%)
Total hypopituitarism	none	3 (14.2%)	none	3 (10.3%)
Diabetes insipidus	none	1 (4.7%)	none	1 (3.4%)
Preoperative Visual Symptoms				
None	3 (100%)	9 (42.8%)	4 (80%)	16 (55.17%)
Monolateral quadrantopia	none	3 (14.2%)	1 (20%)	4 (13.7%)
Bilateral quadrantopia	none	5 (23.8%)	none	5 (17.24%)
Bilateral hemianopia	none	4 (19%)	none	4 (13.7%)

### Extent of resection

In terms of extent of resection, we defined gross total removal a condition of complete cyst content evacuation with cyst wall removal, while subtotal removal was intended as cyst drainage with eventual cyst wall partial removal or biopsy. It is worth reminding that in the present case series, the standard approach was reserved for the treatment of a purely intrasellar or intra and suprasellar cysts, whilst the extended endoscopic surgical route was adopted in those cysts with a purely suprasellar location or to remove the suprasellar component of intra-suprasellar cysts.

Though, we obtained an overall gross total removal in 55.17% of cases (16/29), while a subtotal removal was obtained in 44.83% of patients (13/29).

In patients with intrasellar cyst, we achieved a gross total removal rate of 33.3%, whilst in 66.6% of patients removal was subtotal, due to the adherence of the cyst wall to the normal pituitary tissue.

Gross total removal for intra-suprasellar cysts was 47.6% (10/21), being different in those patients that underwent a standard approach, i.e. 37.5% (6/16), or an extended approach, i.e. 80% (4/5).

In all 5 patients with purely suprasellar RCC, we accomplished a gross total removal (see Table 3).

**Table 3:** Removal rate according to cyst location and surgical approaches.

Location	Intrasellar	Intra and suprasellar		Suprasellar	Total
Surgical approach (n^0^ of cases)	Standard approach (3)	Standard approach (16)	Extended approach (5)	Extended approach (5)	29
Gross total removal	1 (33.3%)	6 (37.5%)	4 (80%)	5 (100%)	16 (55.17%)
Subtotal removal	2 (66.6%)	10 (62.5%)	1 (20%)	none	14 (44.82%)

Concerning reconstruction techniques, they differed according to surgical procedure adopted: in case of standard approaches without any intraoperative CSF leakage no reconstruction was performed (26.3%, 5/19); whether CSF leak had occurred the most used technique was intradural filling with extradural closure (44.8%, 13/29). In case of extended approach the gasket seal closure of the skull base defect supported by the nasoseptal flap was the most common technique used, (60%, 6/10) (see Table 4).

**Table 4:** Reconstruction techniques according to surgical approach.

Reconstruction Technique	Standard approach (19)	Extended approach (10)	Total (29)
None (marsupialization)	5 (26.3%)	none	5 (17.2%)
Intradural filling + intradural closure	2 (10.5%)	none	2 (6.8%)
Intradural filling + Extradural closure	10 (52.6%)	3 (30%)	13 (44.8%)
Intradural filling + Intra/Extradural “gasket seal” closure	none	1 (10%)	1 (3.4%)
Intradural filling + Intra/Extradural “gasket seal” closure + nasoseptal flap	2 (10.5%)	6 (60%)	8 (27.5%)

### Postoperative clinical outcomes

56.2% of patients (9/16) had complete recovery from headache after surgery, whereas the 43.8% (7/16) did not record any improvement.

Concerning the endocrinological status, 5 out of 7 (71.4%) patients with hyperprolactinemia presented the normalization of prolactine levels; condition of partial or total hypopituitarism remained unchanged after surgery except in 2 patients, who experienced a slight improvement of the pituitary deficit (recover of one axis function) (1 out of 5–20%—in partial hypopituitarysm a 1 out 3–33.3% in total hypopituitarism). The patient with diabetes insipidus did not recover from this condition, while 3 new cases (10.3%, 3/28) of DI were observed.

Concerning the visual function, we recorded a postoperative improvement in 53.8% of patients (7/13) and in 38.5% of cases (5/13) complete recovery was observed. We did not detect the onset of any new postoperative visual defect.

### Results according to cyst location and surgical approach

Among the group of patients that underwent standard approach for the treatment of an intrasellar cyst, we did not report any improvement of the pituitary functions.

In the group of patients with intra-suprasellar cysts with the use of a standard approach we achieved the improvement of pituitary function in one case of partial hypopituitarysm as well as the complete recovery in all cases from the condition of PRL hypersecretion. In these lesions the extended approach allowed the improvement of one case complaining of preoperative panhypopituitarysm. Concerning suprasellar lesions, all of them underwent an extended approach that permitted complete recovery of condition of hyperprolactinemia in one of the two cases. In terms of visual function, the standard approach allowed to achieve recover as well as the improvement of six cases among the group of patients with intra-suprasellar lesions. Conversely, the extended approach granted the complete recovery from visual field defect in 2 cases of intra-suprasellar cysts and in a single case of purely suprasellar lesions. Finally, it should be noted that standard approach led to the onset of a postoperative case of DI and three cases of a partial hypopituitarism; the extended approach has been accompanied by the postoperative occurrence of two cases of permanent DI and a single case of anterior pituitary defect. All the data concerning postoperative endocrinological and visual functions are summarized in Table 5.

**Table 5:** Postoperative clinical status according to surgical approach.

Postoperative Endocrinological Status	Standard approach	Extended approach	Total
Normal pre-operative pituitary function (14)			
Unchanged	5 (35.7%)	5 (35.7%)	10 (71.1%)
Worsening	3 (21.4%)	1 (7.1%)	4 (28.5%)
Partial pre-operative hypopituitarism (5)			
Unchanged	3 (60%)	1 (20%)	4 (80%)
Improved	1 (20%)	none	1 (20%)
Worsening	none	none	none
Total hypopituitarism (3)			
Unchanged	2 (66.6%)	none	2 (66.6%)
Improved	none	1 (33.3%)	1 (33.3%)
Diabetes insipidus (1)			
Unchanged	none	1 (3.4%)	1 (3.4%)
New cases	2 (7.1%)	1 (3.5%)	3 (10.6%)
Preoperative hyperprolactinemia (7)			
Unchanged	1 (14.2%)	1 (14.2%)	2 (28.4%)
Improved	4 (57.1%)	1 (14.2%)	5 (71.3%)
Postoperative Visual Status			
Visual defects (13)			
Unchanged	1 (7.6%)	none	1 (7.6%)
Improved	5 (38.4%)	2 (15.3%)	7 (53.8%)
Resolved	3 (23%)	2 (15.3%)	5 (38.4%)
Worsening	none	none	none
New Onset	none	none	none

### Results according to capsular removal

We achieved a gross total removal, i.e. we removed completely the cyst wall in 16 out of 29 patients; among these 5 patients (5/5, 100%) were presenting purely suprasellar lesions and were treated via an extended approach; 10 (10/21, 47.6%) were complaining of an intra-suprasellar lesion: 4 (4/5, 80%) received an extended approach and in 6 cases (6/16, 37.5%) a standard approach was adopted. Gross total removal was achieved via a standard approach also in a single patient (1/3, 33.3%) with intrasellar tumor.

Complete cyst wall removal allowed a complete recovery in 4 patients (4/7, 57.1%), who were complaining headache preoperatively; as well 5 patients (5/9, 55.5%), who received a subtotal removal (cyst drainage with cyst wall partial removal or biopsy) reported a complete recovery from headache.

In terms of visual function, in the group of patients that received gross total removal, we obtained a post-operative improvement in 5 cases (5/8, 62.5%) and in 3 cases (3/8, 37.5%) a total recovery was noted. Conversely, in the group of patients in which we achieved a subtotal removal we had 3 caases (3/5, 60%) of visual defects total recovery, 1 (1/5; 20%) patient improved and 1 (1/5; 20%) remained unchanged. In this latter group the endocrinological stuatus remained unchanged except for 3 patients (3/13, 23.1%) that complained post-operative worsening.

Among the patients who underwent a gross total removal, the endocrinological status was not altered except for a single case (1/3, 33.3%) of intra-suprasellar lesion that improved pre-operative panhypopituitarism and a case of intra-suprasellar tumor (1/16, 6.2%), in which a worsening of the pituitary function was observed.

## Complications

In our series none of patients with purely intrasellar cysts presented complications. The most frequent post-operative complication was represented by diabetes insipidus. 6 patients (20.6%, 6/28) developed a transient diabetes insipidus, 3 patients (10.3%) developed a permanent one and started medical therapy with desmopressine. In 2 cases (6.9%) we observed a post-operative CSF leak, 1 was an intra-suprasellar cysts treated with a standard approach and 1 was a purely suprasellar cyst, treated with an extended approach. Both required a second endoscopic endonasal approach for achieving a proper reconstruction of the skull base defect.

Of these, patient with intra-suprasellar RCC who underwent a second surgery in the 5^th^ post-operative day because of a CSF leak developed a pneumocephalus and a thalamic ischemia in the late post-operative course.

CSF leak occurred in a single case for each apart of the groups of patients that received gross total removal, (1/16, 6.2%) and of the group of patients that underwent subtotal removal (1/13, 7.7%). Transient DI occurred in 4 patients that underwent gross total removal (4/16, 25%) and in 2 cases (2/13, 15.4%) treated with a subtotal removal. Three cases (3/16, 18.7%) of permanent DI were found exclusively in the former group.

The data concerning the complications according to cyst location are reported in Table 6.

**Table 6:** Complications according to cyst location.

Complications	Intra sellar	Intra and supra sellar		Supra sellar	Total (29)
	Standard approach (3)	Standard approach (16)	Extended approach (5)	Extended approach (5)	
Post-op CSF leak	none	1 (6.2%)	none	1 (20%)	2 (6.9%)
Diabetes insipidus transient	none	2 (12.5%)	1 (20%)	3 (60%)	6 (20.6%)
Diabetes insipidus permanent	none	1 (6.2%)	1 (20%)	1 (20%)	4 (13.7%)
Thalamic ischemia	none	1 (6.2%)	none	none	1 (3.4%)

### Recurrence and regrowth

None of the patients that received gross total removal presented recurrence or regrowth at follow-up.

Four cases complaining of intra-suprasellar location, who received a primary subtotal removal (4/13, 30.8%), presented a regrowth of the cyst, with a mean time of appearance after the first surgery of 60 months. One of these, upon becoming symptomatic, underwent a successfully standard approach that resulted in gross total removal. For the rest of patients we observed a stable and asymptomatic regrowth of the cyst at follow up, so that no further surgical treatment has been planned.

## Discussion

Rathke’s cleft cysts (RCCs) are generally asymptomatic or seldom present with several clinical symptoms: headache, pituitary dysfunction, or visual disturbances. Uncommon presentations include pituitary apoplexy, impaired growth, diabetes insipidus and sexual function impairment such as erectile dysfunction [2, 5, 7, 8, 10, 11, 13, 15, 16, 18, 22, 25, 26, 42, 43].

Although headache is the most common presenting symptom, its origin has not yet defined; several authors have claimed a causal role of the mass effect exerted from the lesion over the surrounding structures such as the cavernous sinus, dura mater and its arteries, internal carotid arteries and trigeminal nerve roots [10, 15, 18, 19, 22, 25, 27, 44]. In these regards, same authors highlighted how the cyst decompression resulted in complete relief of headache, with a success rate varying from 32 to 91%. In our series, headache was the presenting symptom in 16 patients (52,1%); 9 (56.2%) had complete pain resolution after surgery and 7 patients did not achieve any improvement.

Concerning the preoperative pituitary dysfunction, our data are consistent with the pertinent literature, where it has been reported a rate of 30–70%: we had 15 of 29 patients (51,17%) with a preoperative pituitary dysfunction.

As reported in the literature, rate of postoperative new-onset hormonal deficits is 4–30% [2, 7, 9, 13, 15, 25, 43]. In our series 4 patients (13,7%) had newly diagnosed hormonal defects.

Again, when analyzing our data, it seems that our outcomes are consistent with previous reports [6, 9, 12, 15, 17, 19, 22–24, 27, 36, 40, 43, 45].

In our experience, the cysts with a purely intrasellar location mostly presented with a single axis dysfunction and no visual defects, while the cysts with an intra and suprasellar location usually presented with a severe hypophyseal and, at least, moderate visual field defect. In purely suprasellar cysts the most common hormonal finding was represented by hyperprolactinemia, and only one patient presented a visual field defect (VFD) (monolateral quadrantopia).

As described in literature, hypophiseal dysfunctions are often accompanied by visual field defects with an incidence of 14% [6, 9, 12, 15, 17, 19, 22–24, 27, 36, 40, 43, 45].

Rathke’s cleft cysts have been traditionally operated through a “standard” transsphenoidal route, with the transcranical route advocated only for those symptomatic lesions located in the suprasellar region. The wide use of the endoscopic endonasal surgery pushed the introduction of this technique, the so called “extended” and its variations for the treatment of different skull base lesions that made possible to gain direct access to the suprasellar area [1, 4, 28–32, 40, 46–51].

The choice of the approach, in our experience, was made upon the evaluation of cyst location [6]: this definition of the surgical strategy pertained mostly the intra-suprasellar lesions. As a matter of fact, all the intrasellar cysts received a standard approach, while all the purely suprasellar RCCs was treated with an extended approach. Out of 21 patients with cysts with an intra and suprasellar location, we performed an extended approach in those cases with a larger suprasellar component and when the transsellar route could damage the pituitary gland or didn’t let to access and/or empty the suprasellar component of the lesion. We aimed to completely drain the colloid and achieve as much as possible cyst wall removal. In these cases, the strict adhesion with the branches of hypophyseal artery going to the pituitary stalk and to the optic chiasm was the limiting factor for a total removal. Contrariwise, in standard procedures our concern was to preserve the hypophyseal function, avoiding hormonal defects; so, when tightly attached to the gland, the cystic capsule was left behind.

A normalization of prolactin levels was noticed after surgery in all patients with hyperprolactinemia, mostly related to the complete relief of the pituitary stalk. In patients with partial or total hypopituitarism this condition remained unchanged, but in two cases an improvement of the pituitary deficit (1 case of partial and 1 case of total hypopituitarism) was observed. The only case of preoperative diabetes insipidus did not improve after surgery. Concerning visual outcome, out of 13 (44.82%) patients with visual field defects (VFDs), 7 (53.8%) had an improvement, while in 5 cases (38.46%) a total resolution was observed. In none of the patient a worsening or a new onset ophthalmologic disease was noticed.

When analyzing clinical outcomes according to the removal rate, namely whether the cyst wall was completely removed or not, we did not found any significant difference between the two groups in terms of both visual (p = 0.3846 at difference of proportions) and endocrinological (p = 0.2994 at difference of proportions) functions.

The most frequent post-operative complication was represented by diabetes insipidus, while the most severe occurred in one patient (3.4%) with intra-suprasellar RCC who underwent a second surgery in the 5th post-operative day because of a CSF leak and consequently developed a pneumocephalus and a thalamic ischemia in the late post-operative course. In regards to the postoperative CSF leakage, we reported an overall 6.9% rate, with 10% of cases occurring in extended approaches and 5.3% in standard approaches; all these lesions were intrasuprasellar cysts and all underwent a total removal. These rates appear little lower when compared to those reported in the pertinent literature for microscopic transsphenoidal approach [2, 5, 9, 10, 13, 15, 24, 25, 28–30, 42].

Concerning the recurrence rates, we noticed that a subtotal removal of the lesions was associated with a higher incidence of recurrence/regrowth as compared to the total removal (p = 0.0752); 4 patients, in which recurrence/regrowth occurred, had undergone a subtotal removal of the cyst, via a standard approach. Nevertheless, total removal had been related to a significant greater overall risk of complications, and this finding has been confirmed by other authors: the study of Fan *et al* [6]found that very aggressive treatment resulted in a higher rate of postoperative DI (42%) compared with less aggressive treatment modalities (9%). In a study by Aho *et al* [45] on a large series of patients with Rathke's cleft cyst, the authors noticed that there was no difference in rate of recurrence between radical and subtotal resections: six of 33 patients undergoing radical resection experienced a recurrence, whereas recurrence occurred in 18 of 85 (p = 0.473) patients who underwent a less radical resection.

Backing upon these data, we retain that the removal of a Rathke's cleft cyst can benefit from the extended variation of the approach in those cases where colloid component cannot be effective drained via a standard transsphenoidal corridor or when the cyst is purely located in the suprasellar area. As well, the cyst wall total removal does not represent a key step to gain the resolution of the pathology or the certain recovery from the preoperative symptoms; it can be though reasonable leave a residual behind when it is tightly attached to the surrounding neurovascular structures. Although we are supported by the evidence of the present series, there is no conclusive evidence that a more aggressive resection of the cyst wall can result in a lower risk of recurrence.

## Conclusion

The endoscopic endonasal approach for the treatment of RCC could be adopted under the same indications of the conventional microsurgical transsphenoidal technique. We found that it presents several advantages in term of visualization of the surgical field during both the exposure and lesion removal. Its “extended” variation provides a direct access to the supradiaphragmatic space, allowing adequate exposure for the removal of supradiaphragmatic RCCs, regardless the size of the sella (even a not enlarged sella), and allows preserving pituitary function. We noted that this technique is safe and effective as we achieved removal rates, risk and complications similar to those presented in large series operated on either via transsphenoidal microsurgical, and/or standard endoscopic endonasal approach.

Proper decision-making process, rigorously founded on a careful preoperative evaluation, as well as on the analysis of the eventual risks and disadvantages is mandatory.

Larger series and longer follow-up are prerequisites to support these preliminary data with further details.
